# The Protective Effects of Astaxanthin on the OVA-Induced Asthma Mice Model

**DOI:** 10.3390/molecules22112019

**Published:** 2017-11-21

**Authors:** Yun-Ho Hwang, Seong-Gyeol Hong, Seul-Ki Mun, Su-Jin Kim, Sung-Ju Lee, Jong-Jin Kim, Kyung-Yun Kang, Sung-Tae Yee

**Affiliations:** 1 College of Pharmacy, Sunchon National University, 255 Jungangno, Suncheon 540-950, Korea; hyh7733@naver.com (Y.-H.H.); hong9217@naver.com (S.-G.H.); motomoto1210@naver.com (S.-K.M.); ksz1353@naver.com (S.-J.K.); 6525140@hanmail.net (S.-J.L.); 2 Singapore Bioimaging Consortium, Agency for Science, Technology and Research, 11 Biopolis Way, No. 02-02 Helios, Singapore 138667, Singapore; joadon09@nate.com; 3 Suncheon Research Center for Natural Medicines, Suncheon 540-950, Korea; kang8404@nate.com

**Keywords:** asthma, airway hyperresponsiveness (AHR), helper T cells (Th cells), astaxanthin

## Abstract

Although astaxanthin has a variety of biological activities such as anti-oxidant effects, inhibitory effects on skin deterioration and anti-inflammatory effects, its effect on asthma has not been studied. In this paper, the inhibitory effect of astaxanthin on airway inflammation in a mouse model of ovalbumin (OVA)-induced asthma was investigated. We evaluated the number of total cells, Th1/2 mediated inflammatory cytokines in bronchoalveolar lavage fluid (BALF) and airway hyperresponsiveness as well as histological structure. The level of total IgE, IgG1, IgG2a, OVA-specific IgG1, and OVA-specific IgG2a were also examined. The oral administration of 50 mg/mL astaxanthin inhibited the respiratory system resistance, elastance, newtonian resistance, tissue damping, and tissue elastance. Also, astaxanthin suppressed the total cell number, IL-4, and IL-5, and increased the IFN-γ in the BALF. In the sera, total IgE, IgG1, and OVA-specific IgG1 were reduced by astaxanthin exposure and IgG2a and OVA-specific IgG2a were enhanced via oral administration of astaxanthin. Infiltration of inflammatory cells in the lung, production of mucus, lung fibrosis, and expression of caspase-1 or caspase-3 were suppressed in OVA-induced asthmatic animal treated with astaxanthin. These results suggest that astaxanthin may have therapeutic potential for treating asthma via inhibiting Th2-mediated cytokine and enhancing Th1-mediated cytokine.

## 1. Introduction

Asthma, one of the allergic diseases, affects 300 million people worldwide. The prevalence of asthma in children and adults increases globally by 50% every decade in different countries. Especially, in developing regions such as Africa, Central and South America, Asia, and the Pacific, the prevalence of asthma continues to rise sharply due to the increasing urbanization and westernization. This increased incidence of asthma leads to an increase in the cost of the treatment [1]. Asthma is a complex syndrome with many phenotypes in humans and is characterized by variable airflow obstruction, airway hyperresponsiveness (AHR), and airway inflammation [2], which cause wheezing, coughing, tightness of the chest, and breathlessness [3].

Asthma is associated with a combination of immunological, genetic and environmental interactions [4]. The inflammatory responses in asthma are linked to the infiltration of the airway wall with a range of inflammatory cells driven in large part by the activation of Th2-type lymphocytes, mast cells and eosinophils [5]. The CD4^+^ T cells are namely divided into Th1 and Th2 cell subsets, and a larger number of Th2 cells than Th1 cells are found in the airways of patients with asthma. The key pathophysiological features of asthma are maintained by IL-4, IL-5, and IL-13 cytokines secreted from Th2 cells. IL-4 has long been recognized as an important cytokine in the development of Th2 cells and is important for allergic sensitization and IgE production, while IL-5 is crucial for eosinophil survival [6]. Moreover, Th2 cytokines induce the differentiation of B cells to plasma cells, and IL-4 promotes the IgG-IgE switch. On the other hand, interferon gamma (IFN-γ) secreted by Th1 cells inhibits the proliferation and differentiation of basophils, mastocytes, and eosinophils [7].

Astaxanthin (3,3′-dihydroxy-β,β-carotene-4,4′-dione) is one of the xanthophyll carotenoids, which is mainly found in microalgae, fungi, complex plants, seafood, flamingos and quail. The United States Food and Drug Administration approved astaxanthin as a feed additive and it was approved for use as a dietary supplement [8]. Carotenoids are widely distributed in marine organisms and play important roles as antioxidants protecting these organisms from injuries caused by free radicals and active oxygen species [9]. Astaxanthin has a variety of biological activities, such as a wound healing effects on full-thickness dermal wounds in mice [10], inhibitory effects on age-related skin deterioration and maintenance effect on skin conditions associated with environmentally induced damage [11], preventive effects against renal tubular oxidative damage [12], protective effects of astrocytes against trauma-induced apoptosis through the inhibition of NKCC1 expression via the NF-κB signaling pathway [13], and neuroprotective effects in a rat model of spinal cord injury [14]. Furthermore, previous studies have also shown that astaxanthin has an anti-inflammatory effect in the phthalic anhydride-induced atopic dermatitis animal model [15].

Recently, several authors have shown that astaxanthin alleviated acute lung injury by inhibiting the inflammatory response, oxidative/nitrative stress, and pulmonary apoptosis, as well as down-regulating NF-κB P65 expression [16]. However, the effect of astaxanthin on asthma as an inflammatory disease has not yet been investigated. The goal of this study was to confirm whether the oral administration of astaxanthin can prevent airway inflammation in ovalbumin induced asthmatic mice or not.

## 2. Materials and Methods

### 2.1. Reagents

Ovalbumin (A5503) and astaxanthin were purchased from Sigma-Aldrich (St. Louis, MO, USA). Purified rat anti-mouse IL-4, purified rat anti-mouse IL-5, purified rat anti-mouse IFN-γ, biotin rat anti-mouse IL-4, biotin rat anti-mouse IL-5, biotin rat anti-mouse IFN-γ purified rat anti-mouse IgE (R35-72), purified rat anti-mouse IgG1 (A85-3), purified rat anti-mouse IgG2a (R11-89), biotin rat anti-mouse IgE (R35-118), biotin rat anti-mouse IgG1 (A85-1), and biotin rat anti-mouse IgG2a (19-5) were purchased from BD Biosciences (San Diego, CA, USA).

### 2.2. Animals

All mice were treated in strict accordance with the Sunchon National University Institutional Animal Care and Use Committee’s (SCNU IACUC) guidelines for the care and use of laboratory animals. All procedures were approved by the SCNU IACUC (permit number: SCNU IACUC-2017-05). Female C57BL/6 mice (7–8 weeks old) were bred and maintained under specific pathogen-free conditions at ORIENT BIO (Seongnam, Korea). The animals were housed at a controlled temperature of 22 ± 2 °C and at 50 ± 5% relative humidity. The mice were housed in polycarbonate cages and fed a standard animal diet with water. All experiments were performed under zoletil/rumpun anesthesia, and all effort was made to minimize suffering.

### 2.3. Sensitization and Provocation of Airway Inflammation with OVA

**Figure 1:**
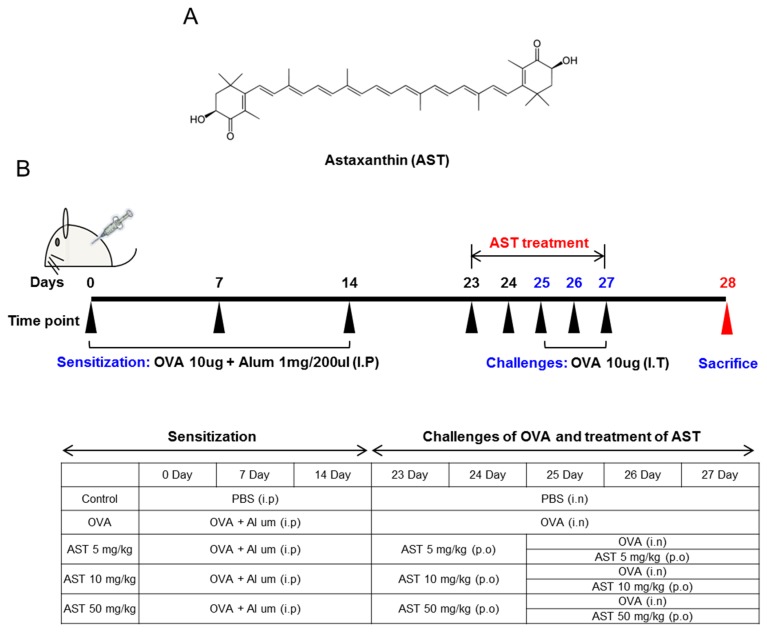
(**A**) The molecular structure of astaxanthin (AST); (**B**) Experimental protocol for the induction and therapy of airway inflammation along with the treatment scheme.

The mice were sensitized and challenged with OVA as previously described [17]. The mice were randomly divided into five groups (*n* = 4): the control group, OVA group, and astaxanthin (at doses of 5, 10 and 50 mg/kg, respectively) + OVA groups. The C57BL/6 mice were sensitized by the intraperitoneal injection of 20 μg of OVA and 100 μg of Imject Alum (Pierce) in 0.2 mL of saline on days 0, 7 and 14. On day 14, the mice were anesthetized and challenged by intranasal instillation of 100 μg OVA in 50 μL phosphate-buffered saline (PBS) or PBS alone for the negative control. On days 25, 26 and 27, the mice were again anesthetized and challenged by the intranasal instillation of 50 μg OVA in 50 μL PBS or PBS alone for the negative control. On days 23–27, astaxanthin (5, 10 and 50 mg/kg) in 0.5% Sodium Tvlose was administered orally once per day (Figure 1B). 24 h after the last airway challenge, blood was collected from the retro orbital plexus. After centrifugation (5000 rpm, 4 °C, 5 min), the serum was stored at −20 °C until assayed for immunoglobulins by ELISA. The animals were sacrificed by cervical dislocation. Bronchoalveolar lavage (BAL) of the mice was performed four times each with 0.5 mL of saline. After centrifugation (1200 rpm, 4 °C, 5 min), the supernatant of BAL obtained from 2 mL of instilled saline was stored at −20 °C until assayed for cytokines by ELISA. The red blood cells in BAL were removed by tris-buffered ammonium chloride. The total cells were counted using a hemacytometer.

### 2.4. Assessment of Airway Hyperresponsiveness on the AST in OVA-Induced Asthmatic Mice

As described in previous studies, we measured the airway hyperresponsiveness (AHR) of the OVA or AST exposed mice (*n* = 2) [18]. Briefly, the final challenged mice were anesthetized using a mixture of Zoletil and Rumpun and the anesthetized mice were tracheostomized using an 18 G metal cannula. The mice was then placed in a flow-type body plethysmograph and connected by the endotracheal cannula to a small-animal ventilator (FlexiVent, SCIREQ Inc., Montreal, QC, Canada). Doses of methacholine (MCh) were administered using a nebulizer (Aeroneb) and progressively doubled concentrations ranging from 0 to 50 mg/mL. The respiratory system resistance (Rrs), respiratory system elastance (Ers), airway resistance (Rn), tissue damping (G), and tissue dynamic elastance (H) were determined before each challenge and after each dose of MCh.

### 2.5. Measurement of Inflammatory Cytokine and Immunoglobulin Production in OVA or AST Exposed Mice

The levels of the various cytokines such as IL-4, IL-5 and IFN-γ in the BAL and immunoglobulins (Ig’s) such as the total IgE, IgG1, IgG2a, OVA-specific IgG1, and OVA-specific IgG2a in the serum were measured by enzyme-linked immunosorbent assay (ELISA).

### 2.6. Histological Analysis of Lung Tissue in OVA or AST Exposed Mice

The left lungs of the mice were removed, transferred into 4% formalin for 24 h (room temperature) and subsequently transferred into PBS. The left lung was dehydrated using ethanol and xylene, embedded in paraffin and 4 μm sections were obtained. The paraffin embedded lung sections were stained with hematoxylin and eosin (H&E), periodic acid-schiff (PAS), and pico sirius red. Images of the lung tissue sections stained with H&E and PAS were acquired with a microscope equipped with a ×20 or ×40 objective lens. For immunohistochemistry, the paraffin-embedded sections were deparaffinized. The slides were washed at room temperature and hydrated. The endogenous peroxidase activity was then quenched with 3% hydrogen peroxidase. The sections were then blocked and the endogenous avidin and biotin were blocked, following the manufacturer’s instructions. The samples were then stained with an antibody against caspase-1 or caspase-3. Biotinylated secondary antibodies (2 μg/mL) were used and were detected with horseradish peroxidase, using a Vectastain Elite ABC (Vector Laboratories). Inflammatory cells/epithelium, PAS positive cells, lung fibrosis and the expression of caspase were analyzed by the Image J program [18].

### 2.7. Statistical Analysis

The results are presented as means ± SDs. Statistical analyses were performed using the SPSS program (SPSS, Chicago, IL, USA). The Student’s *t*-test was used to determine the significances of the differences between the groups. *p* values of *p* < 0.001, *p* < 0.01, or *p* < 0.05 were considered to be statistically significant, as indicated.

## 3. Results

### 3.1. Effects of AST on Airway Hyperresponsiveness (AHR)

**Figure 2:**
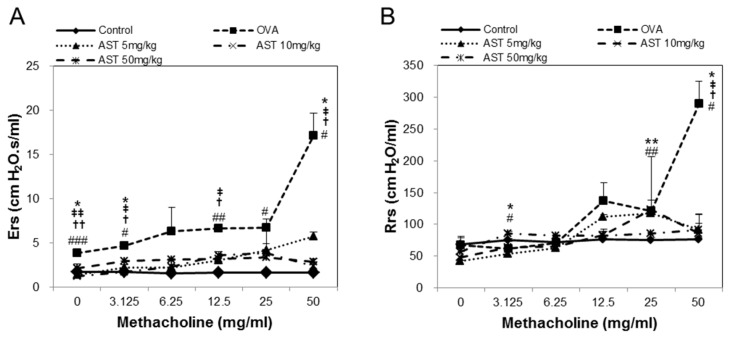

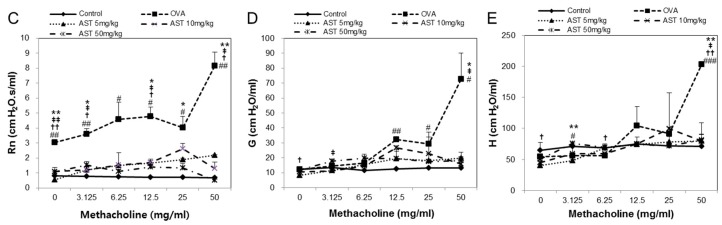
Assessment of allergen-induced airway hyperresponsiveness by the forced oscillation technique. The (**A**) Elastance (Ers); (**B**) Respiratory system resistance (Rrs); (**C**) Newtonian resistance (Rn); (**D**) tissue damping (G); and (**E**) tissue elastance (H) were determined by ovalbumin (OVA) or OVA + astaxanthin (AST). All data were expressed as means ± SD (*n* = 2). # *p* < 0.05, ## *p* < 0.01, and ### *p* < 0.001 control vs. OVA. † *p* < 0.05 and †† *p* < 0.01 OVA vs. AST 5 mg/kg. ‡ *p* < 0.05 and ‡‡ *p* < 0.01 OVA vs. AST 10 mg/kg. * *p* < 0.05 and ** *p* < 0.01 OVA vs. AST 50 mg/kg.

To determine the effect of astaxanthin (AST) on their airway function, the mice were exposed to MCh aerosols. There was no significant difference between the groups at the baseline as regards the total respiratory system resistance (Rrs), elastance (Ers), newtonian resistance (Rn), tissue damping (G), or tissue elastance (H). The Rrs, Ers, Rn, G and H to MCh in the OVA group were significantly higher than those in the control group. This upregulation of Rrs, Ers, Rn, G, and H was reversed by the treatment of AST (Figure 2). These results indicated that AST suppresses AHR in OVA-induced asthma mice.

### 3.2. Effects of AST on Cytokines and BAL Total Cells in OVA-Induced Asthmatic Mice

**Figure 3:**
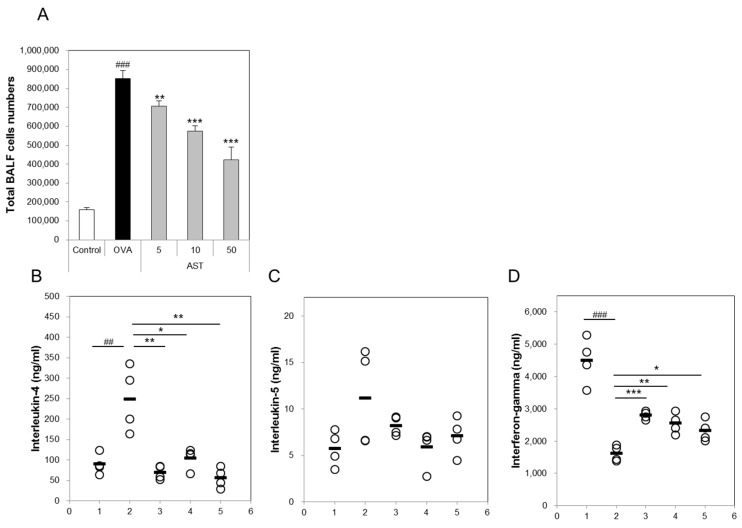
Effects on total cells and Th2-mediated cytokines of AST in BALF. C57BL/6 mice were sensitized and challenged with OVA and AST. In these mice, BALF were collected 24 h after the last challenge. (**A**) The total BALF cells were counted by trypan blue staining; The levels of (**B**) IL-4; (**C**) IL-5; and (**D**) IFN-γ in BALF were measured by ELISA. The data represent four mice per group. ‘−’ indicates the mean of four mice. ## *p* < 0.01 and ### *p* < 0.001 control vs. OVA. * *p* < 0.05, ** *p* < 0.01, and *** *p* < 0.001 OVA vs. AST. Group numbers (1 = control, 2 = OVA, 3 = AST 5 mg/kg, 4 = AST 10 mg/kg, 5 = AST 50 mg/kg).

The present study aimed to investigate whether AST modulates the Th1/Th2 cytokine balance in mice. To confirm the expression of the Th1 or Th2 mediated cytokines, we measured the numbers of IL-4, IL-5, IFN-γ, and total bronchoalveolar lavage fluid (BALF) cells. The total number of BALF cells and IL-4 in the OVA group was increased in comparison with that in the control group. The treatment with AST significantly decreased the concentrations of IL-4 and the BALF total cell numbers (Figure 3A,B). In contrast, the IFN-γ level decreased in the OVA treated group and was upregulated by the treatment with AST (Figure 3D). However, there was no significant difference in the levels of IL-5 between the OVA and AST groups (Figure 3C).

### 3.3. Effects of AST on the Release of Immunoglobulins in Serum

**Figure 4:**
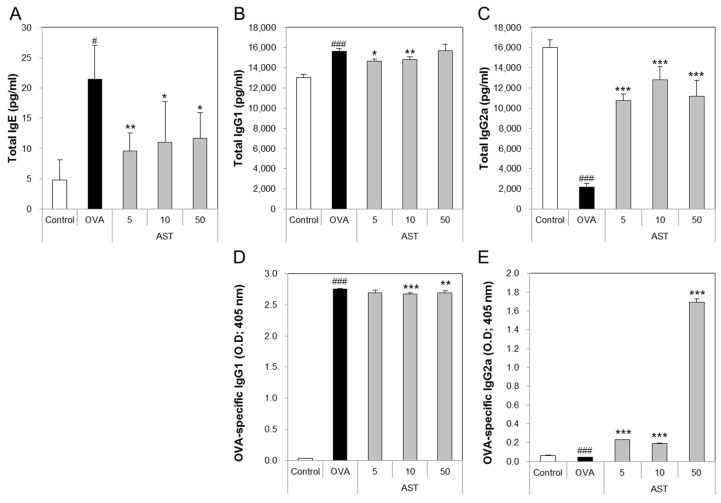
Effects on total cells and Th1 and Th2-mediated immunoglobulins of AST in serum. C57BL/6 mice were sensitized and challenged with OVA and AST. In these mice, serum were collected 24 h after the last challenge. (**A**) The total IgE; (**B**) IgG1; (**C**) IgG2a; (**D**) OVA-specific IgG1; and (**E**) OVA-specific IgG2a were measured by ELISA. The data represent four mice per group. All data were expressed as means ± SD. # *p* < 0.05 and ### *p* < 0.001 control vs. OVA. * *p* < 0.05, ** *p* < 0.01, and *** *p* < 0.001 OVA vs. AST.

The levels of total IgE, IgG1, and OVA-specific IgG1 in the OVA group were significantly increased compared with the control group. The treatment of the OVA group with AST significantly suppressed the levels of total IgE, IgG1, and OVA-specific IgG1 compared with the OVA group (Figure 4A,B,D). In contrast, the levels of total IgG2a and OVA-specific IgG2a in the OVA group were lower than those in the control group. The exposure of the OVA-induced asthma mice to AST increased the total IgG2a and OVA-specific IgG2a (Figure 4C,E). These results suggest that AST suppresses the levels of the IgG’s (IgE and IgG1) associated with the Th2 response and increases the level of IgG2a associated with the Th1 response.

### 3.4. Effects of AST on Histological Changes in Asthmatic Mice

**Figure 5:**
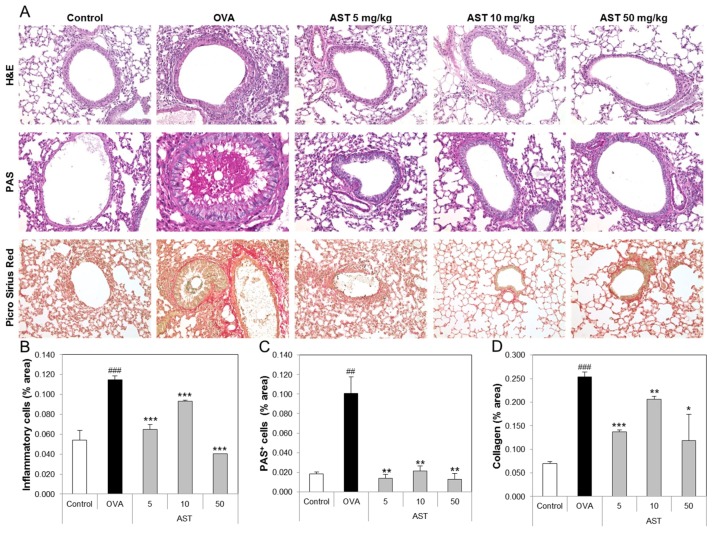
Effect of AST on histology of lung tissue in OVA-induced murine model of asthma. The C57BL/6 mice were sensitized and challenged with OVA for asthma induction. At the end of the experiment, the mice lungs were removed. (**A**) The lungs were stained by H&E (×200), PAS (×200), and Picro Sirius Red. The percentages of (**B**) inflammatory cells, (**C**) PAS positive cells, and (**D**) collagen in the lung sections were measured via Image J program. The data represent three mice per group. All data were expressed as means ± SD. ### *p* < 0.001 and ## *p* < 0.01 control vs. OVA. * *p* < 0.05, ** *p* < 0.01 and *** *p* < 0.001 OVA group vs. AST group. H&E: hematoxylin-eosin staining, PAS: Periodic acid-Schiff staining.

**Figure 6:**
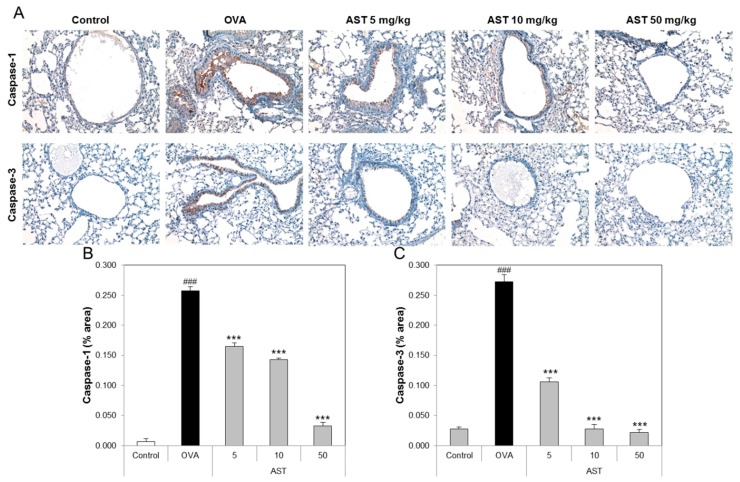
Effect of AST on immunohistochemistry in lung tissue of OVA-induced murine model of asthma. C57BL/6 mice were sensitized and challenged with OVA for asthma induction. At the end of the experiment, the mice lungs were removed. (**A**) The lungs were stained by caspase-1 (×200) and caspase-3 (×200) immunohistochemistry. The percentages of (**B**) caspase 1 or (**C**) caspase 3-positive cells in the lung sections were measured via Image J program. All data were expressed as means ± SD. The data represent three mice per group. ### *p* < 0.001 control vs. OVA. *** *p* < 0.001 OVA group vs. AST group.

To determine the effect of AST on the lung tissue of the asthmatic animals, we performed tissue staining, such as H&E, PAS, and Pricosirius Red (Figure 5A). The inflammatory cell infiltration and epithelial thickening experiment revealed that the inflammatory cell infiltration of the OVA-induced asthmatic mice group was increased compared to that of the control group. However, the exposure of the asthmatic mice to AST decreased the infiltration of inflammatory cells in the airway epithelium of the AST exposed mice group (Figure 5B). To observe the interstitial goblet cell hyperplasia, we performed PAS staining. The number of PAS positive cells in the OVA group was higher than that in the control group. On the other hand, the exposure of asthmatic mice to AST decreased the incidence of goblet cell hyperplasia (Figure 5C). Furthermore, the increased collagen deposition induced by the OVA treatment in the mice was reduced by the exposure to AST (Figure 5D). Further evidence of lung inflammation was provided by the increased levels of Caspase-1 and Caspase-3 in the OVA group compared to the control group. However, the exposure of the OVA-induced asthmatic mice to AST decreased the expression of Caspase-1 and Caspase-3 (Figure 6). These results indicated that the AST suppresses lung inflammation, mucus production, and lung fibrosis in OVA-induced asthma mice.

## 4. Discussion

Since the 1980s, the asthma mortality rate has been progressively reduced around the world, though it would be even lower if the recommended guidelines, which suggest that better implementation of the established management strategies is needed, were followed. However, novel strategies will also be required to achieve a further substantive reduction in the asthma mortality rate [19]. Strategies for treating asthma include the inhalation and oral administration of corticosteroids, leukotriene modifiers, theophyline and anti-IgE and specific allergen immunotherapy (AI). AI is a method of decreasing the ratio of the Th2 to Th1 response or increasing the proportion of Treg cells (regulatory T cells) and, as such, it is effective in treating asthma [20]. In the present study, we conducted experiments based on the Th1 and Th2 responses.

The macrophages activated by the IL-4, IL-13, and IL-33 responses release IL-4 and IL-13 during the allergic airway inflammatory response. These cytokines stimulate the allergic asthma by activating Th2 cells [21]. Astaxanthin, a well-known antioxidant, inhibited the signaling cascade of proinflammatory gene expression in LPS-stimulated macrophages by suppressing NF-κB activation [22]. Furthermore, the phytohemaglutinin (PHA)-induced expansion of CD3^+^CD25^+^ subpopulation (T cells) in the asthmatic patients was significantly suppressed by their exposure to astaxanthin in the in vitro experiment [23]. Although astaxanthin has been reported to inhibit activated macrophages and T cells, the effects of astaxanthin on asthmatic animal models have not yet been tested. Based on the experiments conducted in the present study, we identified the therapeutic effects of astaxanthin in asthmatic mouse models.

Airway hyperresponsiveness (AHR) is known to be associated with both the development and remission of respiratory symptoms in the adult general population and is one of the key pathophysiological features of asthma [24,25]. In previous studies, we confirmed that various AHR factors, such as the respiratory system resistance (Rrs), respiratory system elastance (Ers), central airway resistance (Rn), lung tissue damping (G), and lung tissue elastance (G), were increased in OVA-induced asthmatic mice [18]. In this study, we demonstrated that astaxanthin suppresses AHR, suggesting its potential as a therapeutic agent for asthma.

Interleukin (IL)-4 is a key cytokine in the development of allergic asthma and has the ability to drive the differentiation of naive T helper type 0 (Th0) lymphocytes into Th2 lymphocytes. IL-4 secreted from Th2 cells induces the ε isotype switch and the secretion of immunoglobulin E (IgE) by B lymphocytes [26]. IgE stimulates mast cells and these activated mast cells release histamine and express a number of inducible genes, including IL-4. In other words, activated mast cells affect the dermal allergic responses, as well as asthma [27]. Moreover, IL-5 is associated with the production, mobilization, activation, recruitment, proliferation, survival and suppression of apoptosis in eosinophils at the site of inflammation. Anti-IL-5 therapy is an effective way to manage hypereosinophilic syndrome (HES) patients [28]. In contrast, interferon-gamma (IFN-γ) secreted by Th1 cells plays an important role in Th1 differentiation and exerts direct inhibitory effects on Th2 cytokine production (IL-4 and IL-5) and Th2 proliferation [29]. T-bet is a transcription factor that induces the differentiation of naive CD4^+^ T cells into Th1 cells. T-bet deficient mice exhibited various features and pathological characteristics of asthma. T-bet might be an attractive target for the development of anti-asthmatic drugs [30]. IFN-γ and IL-4 secreted from T cells have potent effects on B cell proliferation and differentiation. IFN-γ stimulates the expression of IgG2a isotype and inhibits the production of IgG3, IgG1, IgG2b, and IgE. In contrast, IL-4 has powerful effects ranging from promoting switching to the expression of IgGl [31]. In the present study, we demonstrated that astaxanthin suppresses the levels of IL-4, IL-5, and IgG1 and enhances the levels of IFN-γ and IgG2a.

The inflammatory cells, such as eosinophils, CD4 T lymphocytes and mast cells, were accumulated in the airway wall (between the smooth muscle and the basement membrane). The increase in the smooth muscle mass, mucous gland hypertrophy and vascular congestion led to the thickening of the airway wall and reduction of the airway caliber. These features may affect the development of airflow limitation by increasing the airway resistance [32]. IL-4 as a pro-fibrotic cytokine is elevated in radiation-induced pneumonitis and pulmonary fibrosis, as well as in liver fibrosis [33]. Collagen accumulation is a major feature of pulmonary fibrosis [34]. We showed that astaxanthin inhibits the infiltration of inflammatory cells, mucus production and lung fibrosis. The cysteine protease, caspase-1 (Casp-1), is an endogenous cysteine protease synthesized as inactive pro-caspase-1 and activated by dimerization and autoproteolysis within inflammasomes, and activated Casp-1 plays a key role in inflammation [35]. Caspase-3 plays an important role in changing pro-IL-16 to the mature cytokine and IL-16 affects allergen-induced airway hyperreactivity and the up-regulation of IgE [36]. In this study, we confirmed that astaxanthin suppresses the expression of caspase-1 and caspase-3 in the lungs of OVA-induced asthmatic mice.

## 5. Conclusions

In conclusion, all of the data in this experiment indicated that astaxanthin has a protective effect against OVA-induced allergic asthma mice. The exposure of the mice to astaxanthin attenuated their airway inflammation and reduced the levels of IgE and IgG1. In addition, astaxanthin can regulate the Th1/Th2 imbalance by inhibiting the release of Th2 cytokines (IL-4 and IL-5) or increasing the release of Th1 cytokines (IFN-γ). Moreover, astaxanthin effectively suppressed AHR, the infiltration of inflammatory cells in the lung, mucus hypersecretion, lung fibrosis, and the expression of caspase-1 and caspase-3. Therefore, astaxanthin could be a promising protective agent for asthma patients.
